# Editorial: Advances in Research on Age in the Workplace and Retirement

**DOI:** 10.3389/fpsyg.2017.02147

**Published:** 2017-12-11

**Authors:** Cort W. Rudolph, Hannes Zacher, Susanne Scheibe

**Affiliations:** ^1^Department of Psychology, Saint Louis University, St. Louis, MO, United States; ^2^Institute of Psychology, Leipzig University, Leipzig, Germany; ^3^Department of Psychology, University of Groningen, Groningen, Netherlands

**Keywords:** editorial, aging, work, retirement, organizational psychology

The global workforce is aging at an unprecedented rate, resulting in changes to the structure and processes of organizations that have redefined how we understand working and retirement (Rudolph et al., 2018). Modern career pathways and the changing nature of working have each contributed to the emergence of distinct forms and patterns of work experiences across the prototypical work lifespan(Wang and Wanberg, 2017). Likewise, older individuals are increasingly delaying retirement in favor of extended labor force participation(Zhan, 2016). Such changes to the workforce, coupled with dynamic (re)definitions of retirement are important concerns for researchers, practitioners, and policy makers alike, as each can profoundly influence the experience of working across one's career(Beehr and Bennett, 2015). The study of age and work, as well as the study of retirement transitions has recently gained traction, owing in part to the aforementioned trends, and in part to the emergence of evidence that age-related processes are important predictors of various work outcomes at different organizational levels.

In this *Frontiers Research Topic*, our stated goal was to curate a collection of papers that are representative of current trends and advances in thinking about and investigating the role of age in workplace processes and the changing nature of retirement. Our hope in doing so, was to showcase various contemporary ideas and rigorous empirical studies as a means to inform broader thinking and to support enhanced theorizing and organizational practice regarding these processes. We are very pleased to report that we have achieved this goal.

Beginning in August of 2016, we solicited proposal abstracts and additionally considered open-call papers until May of 2017. As a result of these efforts, we received 22 full-manuscript submissions (17 invited from abstracts; 5 received via open call), of which 5 were ultimately rejected (22.72% rejection rate). Of the 17 papers accepted for this research topic, a majority (15) represent empirical contributions, although we also feature two conceptual and literature review papers. These 17 manuscripts were authored by researchers representing a variety of different disciplines (e.g., industrial, work, and organizational psychology, management, human resources, occupational medicine, neuroscience) from a globally-diverse array of regions (Europe, Asia, North America, and Australia). The manuscripts further feature a varied array of methodologies (e.g., experimental designs, policy capturing methodologies, observational designs, longitudinal designs, systematic reviews). These diverse perspectives represent the breadth and scope of research concerning age, working, and retirement; this is indeed a vibrant international and multidisciplinary area of inquiry.

Broadly, the research presented here highlights four important points. First, there are a number of person changes in abilities and motivational factors associated with age (Faber and Walter; Henry et al.) that may impact on aging workers' social preferences (Gärtner and Hertel) and lead them to respond to work conditions differently from young workers (Brienza and Bobocel). Second, apart from general age-related trends in abilities and motivation, there is accumulating knowledge on the active role that workers play in managing their career development over time to facilitate “aging successfully” at work (Le Blanc et al.; Müller and Weigl; Wong and Tetrick). At the same time, we are starting to learn about organizational practices and interventions that support successful aging at work (Kensbock et al.; Oltmanns et al.). Third, hiring processes appear to be more subjected to age discrimination than any other organizational practices, yet the extent of age discrimination depends on how applicants present themselves (Derous and Decoster), decision-makers' personal characteristics (Fasbender and Wang), and the assumptions that decision makers hold about older workers (Kaufmann et al.). Moreover, and to a related degree, the attractiveness of particular job features to job applicants changes with age (Zacher et al.). Finally, researchers are making major strides toward improving our evidence-based knowledge of the retirement transition, and the multi-level factors (e.g., income, leadership, planning) that facilitate or hinder workers' motivation to continue working and their well-being once retired (Davies et al.; Lindwall et al.; Wöhrmann et al.; Yeung and Zhou). We next briefly summarize the studies included in our research topic based upon these important points.

## Person changes with age

Brienza and Bobocel investigate how older workers react to perceptions of justice in their current workplace in two empirical studies. Data from two samples of employees (total *N* = 377) were collected using online panels. The researchers find that employee age moderates the negative relationships of justice perceptions with deviance and emotional exhaustion. Moreover, emotional exhaustion mediates the differential effects of justice perceptions on deviance, and these effects are dependent upon employee age, such that older workers appear to be more sensitive to informational and interpersonal justice, and younger workers appear to be more sensitive to distributive and procedural justice.

Faber and Walter study the capability to correctly recognize collective emotion expressions (i.e., “emotional aperture”), and model the joint roles of age and agreeableness for predicting this ability. This study uses a sample of *N* = 181 German participants between 18–72 years of age who participated in an online study. Results suggest that, among individuals with lower agreeableness, there is a curvilinear relationship between age and emotional aperture. This finding suggests that the emotional aperture of those with low agreeableness peaks in middle adulthood, whereas the emotional aperture of those with high agreeableness is relatively high irrespective of age.

Gärtner and Hertel apply socioemotional selectivity theory to predict that familiar teams are prioritized when occupational future time perspective (OFTP) is perceived to be limited. They test their hypotheses using a within-person online vignette study. *N* = 454 participants were asked to choose between a familiar and a new team in three consecutive trials under various levels of manipulated OFTP. In the control condition (i.e., OFTP not manipulated), higher age indirectly predicts a higher preference for familiar teams through reduced OFTP. Moreover, experimentally restricting OFTP increases the preference for a familiar team over a new team regardless of workers' age.

Henry et al. offer a systematic review and critical discussion of the literature on domain-general future time perspective (*K* = 17 studies) and OFTP (*K* = 16 studies), and highlight implications for future research and practice (Note: Richard E. Boyatzis served as action editor of this article). Considering the broad implications of this review, it is clear that future time perspective at work is an important variable to the study of work and aging. For example, future time perspective can both mediate and moderate relationships between individual and contextual antecedents and occupational well-being, as well as motivational and behavioral outcomes.

## Successful aging at work

Kensbock et al. integrate organizational change and accommodations literatures to propose a theoretical framework of the potential for negative experiences during the job accommodation process. This framework is then applied to a qualitative study with *N* = 73 manufacturing workers participating in a job accommodation program at a German industrial company. Results suggest that problems associated with health-related impairments are mostly solved by accommodations, however employees with disabilities report interpersonal problems and conflicts that are similar to those typically occurring during organizational change (e.g., lack of social support; poor communication). Furthermore, the findings of this research suggest that discrimination, bullying, and maltreatment are common during accommodation processes.

Le Blanc et al. propose an approach to understanding the popular concept of sustainable employability that is based on the ability-motivation-opportunity (AMO) framework. This study uses four different conceptualizations of aging at work to bolster evidence for both the convergent and divergent validity of this framework. Data were collected from *N* = 180 employees in Dutch public service organizations using an online survey. The results show that the four conceptualizations of aging are differently related to the three indicators of sustainable employability proposed within the AMO framework. Noteworthy additional findings are that “organizational age” (or tenure) has the strongest negative relationship with the motivation to continue working, and “functional age” (low work ability) has the strongest negative relationship with the opportunity to continue working.

Oltmanns et al. hypothesize that the recurrent experience of novelty at work is an important condition for brain plasticity. Using a case-control design across a time window of 17 years, this study investigates the effect of recurrent exposure to work-task changes on gray matter volume and cognitive functioning in a sample of middle-aged production workers who have otherwise low levels of job complexity. The results suggest that work task changes are associated with better processing speed and working memory as well as with larger gray matter volume in those brain regions that have been associated with learning, and that typically show pronounced age-related declines. As such, this study offers that recurrent novelty at work could serve as an *in vivo* intervention that counteracts the long-term effects of low job complexity.

Müller and Weigl explore associations between employees' use of selection, optimization, and compensation (SOC) strategies at work and their peer-rated organizational citizenship behaviors (OCB). Using a cross-sectional design with multi-source data, a sample of primary school teachers were sampled (*N* = 114) who reported on their SOC strategy use, while their teaching partners reported on their OCB. Results suggest a positive relationship of loss-based selection behaviors with peer-rated OCB regardless of age. Moreover, there is a positive relationship of compensation behavior with peer-rated OCB for older workers, but the effect is negative for young workers, suggesting that compensation behaviors may be beneficial only at higher ages.

Wong and Tetrick build upon the lifespan theory of control and present a conceptual overview of job crafting as a mechanism for maintaining person-job fit across time. This paper argues that job crafting can be a particularly valuable mechanism for older workers to realign and enhance their demands-abilities and needs-supplies fit by proactively exerting personal agency to make changes to the task, social, and cognitive aspects of their jobs.

## Hiring older workers

Derous and Decoster apply job market signaling theory to investigate whether older applicants benefit from concealing explicit age signals on their resumes (e.g., date of birth) and whether subtle age cues on resumes (e.g., older-sounding names) affect older applicants' hirability ratings. Using an experimental design and a sample of *N* = 610 human resource professionals, the results offer evidence for hiring discrimination of older applicants based on implicit age cues that exist in their resumes, and this effect is more pronounced among relatively older raters. Moreover, concealing one's date of birth led to overall lower ratings compared to not concealing one's date of birth.

Fasbender and Wang use theories of planned behavior and core self-evaluations to explore the direct impact of negative attitudes toward older workers on hiring decisions, and the moderating role of decision-makers' core self-evaluations on this relationship. These relationships were tested using an experimental vignette methodology and a sample of *N* = 102 participants working in human resource management. Results suggest that negative attitudes toward older workers have a consequent negative influence on the desire to hire older people. Moreover, decision-makers' core self-evaluations are found to buffer the relationship between attitudes toward older workers and hiring outcomes.

Kaufmann et al. present the results of two experimental studies of the role of facial appearance and impressions of fitness (i.e., physical and cognitive) on hirability assessments. For both studies, results indicate that older-looking job candidates receive lower hirability ratings, and this can be explained by less favorable fitness impressions. The first study also shows this biasing effect is to some degree mitigated when job candidates offered counter-stereotypic information about their fitness. Additionally, in the second study, facial age-based discrimination was less prevalent for jobs with less costumer contact.

Zacher et al. use an experimental policy-capturing design to test integrative hypotheses derived from job design and lifespan developmental theories (Note: Elias Kapoutsis served as action editor of this article). A sample of *N* = 82 employees indicated their job attraction for each of 40 hypothetical job descriptions in which four job characteristics (i.e., job autonomy, task variety, task significance, and feedback from the job) were systematically manipulated. Results demonstrate that the positive effects of task variety, task significance, and feedback from the job are stronger for younger compared to older employees, whereas there are no significant age-differential effects of job autonomy on job attraction.

## Retirement transitions

Davies et al. draw upon comparative theories of retirement attitudes, offering a model of the direct relationship between job satisfaction and intended retirement age, and an indirect relationship between job satisfaction and intended retirement age, via retirement attitudes. These relationships are also proposed to be conditional upon household income. Data from *N* = 590 workers aged 50 and over from the United Kingdom were collected, and a conditional process analysis was used to test this model. Among other interesting findings, results suggest that higher job satisfaction among average and low household income workers is likely to make the prospect of retirement less attractive. Among high household income workers, however, no indirect effects of job satisfaction are found.

Lindwall et al. introduce the HEalth, Ageing and Retirement Transitions in Sweden (HEARTS) study, and present initial results from the two first waves of this ambitious project. The HEARTS study is an annual effort to study psychological health in the years before and after retirement, as well as both change and stability patterns related to retirement. Results from the first and second waves presented by this paper show that individuals who retired between these waves demonstrate more positive changes in psychological health compared with those who are still working or have previously retired.

Wöhrmann et al. investigate the relationship between respectful leadership and older workers' desired retirement age, and investigate both mediating (i.e., job satisfaction, subjective health, and work-to-private life conflict) and moderating (i.e., occupational self-efficacy) factors that might help to explain the assumed relationships with respectful leadership. A hypothesized model was tested within a large (*N* = 1,130) sample of blue and white-collar workers between 45 and 65 years of age. The results suggest that respectful leadership is positively related to older workers' desired retirement age and that this relationship is mediated by subjective health and lower work-to-private life conflict.

Yeung and Zhou explore the mechanisms underlying the relationship between retirement planning activities and post-retirement well-being. This study adopts a resource-based dynamic model, and uses a longitudinal study design to examine whether pre-retirement planning activities can increase the total resources of retirees (i.e., tangible, mental, and social resources), and consequently contribute to better well-being following retirement. Using a sample of *N* = 118 Hong Kong Chinese surveyed across three time points spanning 6 months prior to retirement until 12 months thereafter, the study suggests positive changes in well-being for retirees who had increases in retirement resources before retirement. Additionally, retirees with more retirement preparatory activities before retirement acquire greater resources initially, which contributes to positive changes in post-retirement well-being over time.

## Conclusion

We hope that this collection of papers inspires researchers to think differently about the study of aging and work, and retirement. When we initiated our call for papers we specified different directions of research that we regarded as important to advance knowledge on the role of age in the workplace and the changing nature of retirement. In reviewing the set of studies included in this research topic, it becomes evident that many of these research directions were adopted by these manuscripts, and that through these collective efforts, we are beginning to see a comprehensive and sophisticated picture of the opportunities and challenges of an aging workforce.

At the same time, the predominant focus of this set of studies still is on individual worker outcomes. Moving the focus from the individual worker to the level of dyads (i.e., vertical or horizontal; e.g., age differences between employees and their supervisors, couples retiring together versus apart), teams (e.g., the role of age diversity), and organizations (e.g., climate for successful aging, role of organizational support in the retirement transition) in future research will be fruitful to fully understand the broader implications of an aging workforce and changing nature of retirement.

The papers included in this research topic have adopted a broad variety of perspectives and methodologies, ranging from theory development papers to systematic reviews, and from qualitative studies and experimental studies to large-scale longitudinal studies. It is laudable that most empirical studies included in this research topic addressed at least one of the recently proposed methodological recommendations to move research on work and aging forward(Bohlmann et al., 2017). For instance, several studies make use of experimental and longitudinal research designs, examine mediators and moderators of relationships between age and work outcomes, and hypothesize and test curvilinear age patterns. At the same time, further research is needed that adopts a multilevel perspective on aging at work and retirement, with multiple predictors and/or outcomes at the individual, dyadic/team, and organizational levels of analysis.

The papers included of this research topic also incorporated a broad range of theoretical traditions. In line with recent suggestions to adopt lifespan perspectives to study work and aging, and retirement(Rudolph, 2016), we see a variety of lifespan theories represented among these studies, from socioemotional selectively theory and the meta-theory of selection, optimization, and compensation, to lifespan theories of control and job design. These perspectives reflect, to some degree, the breadth of theoretical coverage represented here. That said, we also note that research tends to broadly adopt these perspectives, while often not fully elaborating on the predictions derived therefrom. That is to say, it is relatively easy to invoke lifespan perspectives as broad explanations for aging and work or retirement processes. However, the tenets of such theories are rarely tested explicitly by such studies, and the specific developmental mechanisms considered within each theory are often neither operationalized nor observed. More precise mapping of theories onto research is an important consideration for future work in this field.

Last, but not least, we would like to sincerely thank the following reviewers and *ad hoc* action editors (listed below, in alphabetical order) for investing their time and efforts to provide helpful and constructive feedback to the authors of the papers included in this research topic: Neal Ashkanasy, Richard Boyatzis, Jeanette Cleveland, Catherine S. Daus, Ellen Dingemans, Joanne Earl, Gwen Fisher, Marco Giannini, Gabriele Giorgi, Joseph Goodman, Ilke Inceoglu, Ilias Kapoutsis, Dorien Kooij^*^, Yihao Liu, Justin Marcus, Jean McCarthy, Andreas Müller, Michael North, Silvia Profili, Jacob Shane, Christian Stamov Roßnagel, Gregory Thrasher, Gabriela Topa^*^, Matthias Weigl, Johannes Wendsche, Anne Marit Wöhrmann, Kevin Wynne, Dannii Yeung^*^, Sara Zaniboni^*^, Yujie Zhan (Note: ^*^A special thanks is extended to these reviewers who reviewed two or more manuscripts!).

## Author contributions

All authors listed have made a substantial, direct and intellectual contribution to the work, and approved it for publication.

### Conflict of interest statement

The authors declare that the research was conducted in the absence of any commercial or financial relationships that could be construed as a potential conflict of interest.
